# Man With Leg Pain

**DOI:** 10.1016/j.acepjo.2024.100023

**Published:** 2025-01-10

**Authors:** Alejandro Avila, Riley Putnam, Ellen Shank

**Affiliations:** 1 Department of Emergency Medicine, Kaiser Permanente, Fresno, California, USA; 2 Department of Biology, Point Loma Nazarene University, San Diego, California, USA; 3 Department of Emergency Medicine, University of California Davis Medical Center, Sacramento, California, USA

**Keywords:** trench foot, nonfreezing cold injury, homelessness, bullae, emergency department

## 1 Case Presentation

**Figure 1:**
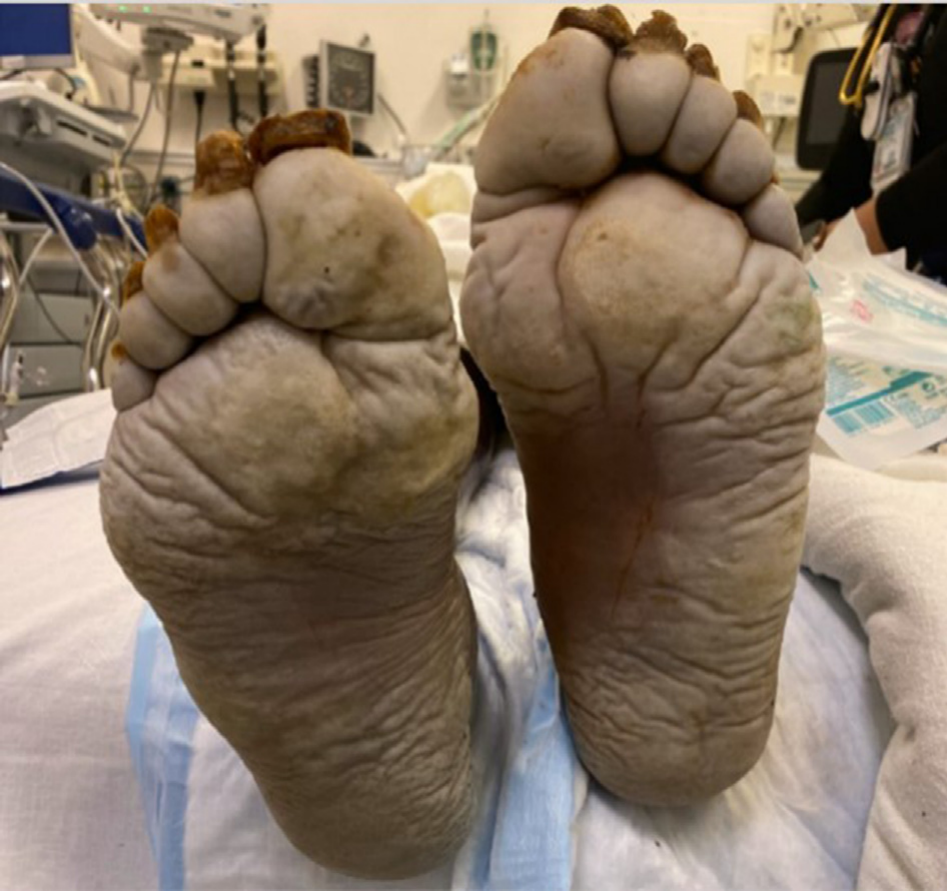
Pallor to bilateral feet on initial presentation.

**Figure 2:**
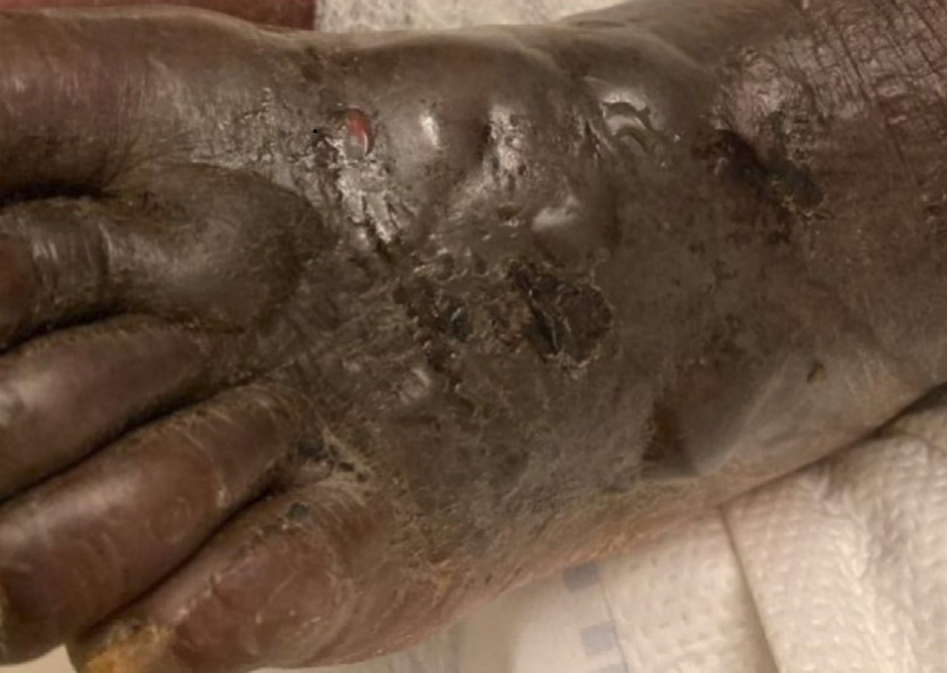
Developing bullae to the dorsum of the left foot.

A 58-year-old unhoused man was brought by ambulance to the emergency department (ED) with leg pain. He had been found lying on the ground barefoot in the rain and was COVID-19–positive. He had a shallow-based ulcer on his left leg and pale feet (Fig 1), with developing bullae noted on the dorsum of bilateral feet (Fig 2).

## 2 Diagnosis: Nonfreezing Cold Injury (or Trench Foot)

**Figure 3:**
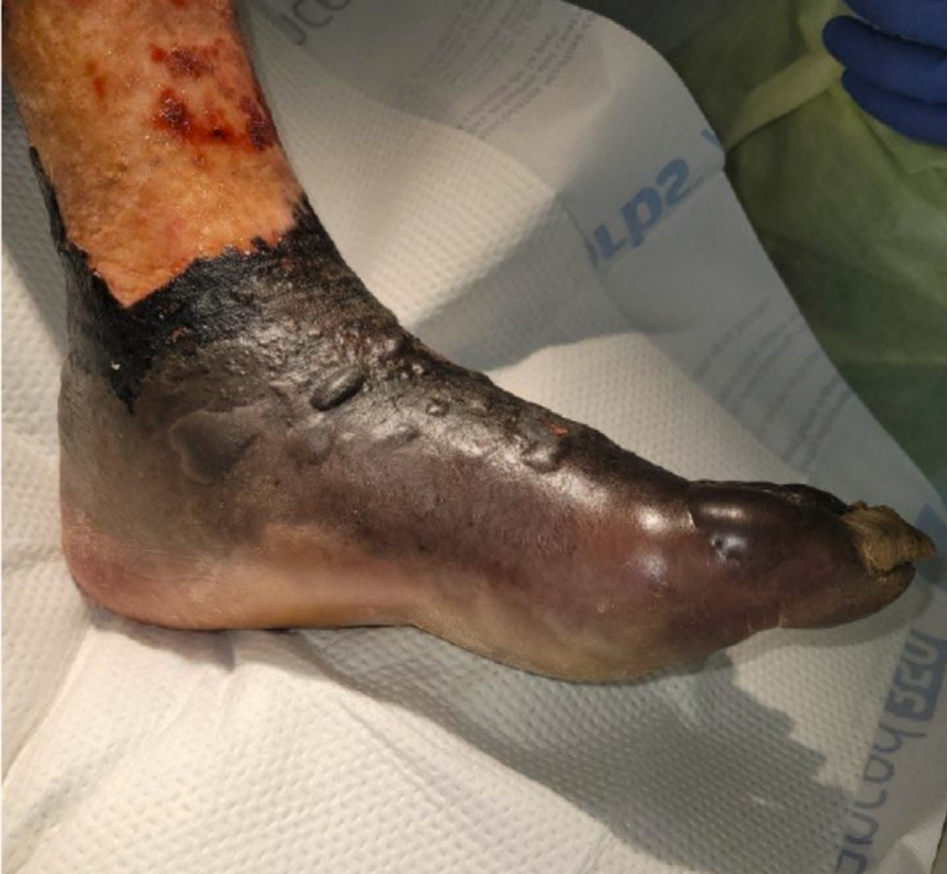
Hyperemia and bullae of the left foot.

**Figure 4:**
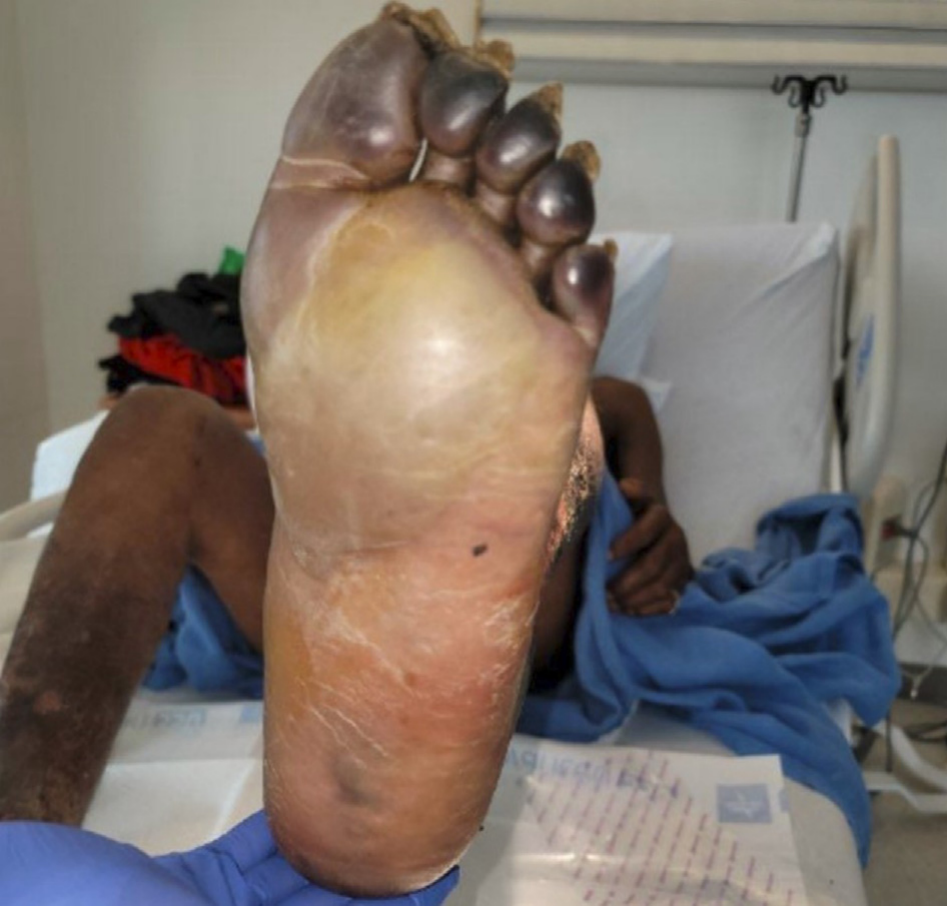
Firm bulla to the sole of the left foot.

Nonfreezing cold injury (NFCI) was considered the most likely diagnosis. The differential included cellulitis, necrotizing soft tissue infection, and COVID-19 toes. Broad-spectrum antibiotics were initiated to cover for *Pseudomonas aeruginosa*; cultures showed no significant growth. Gabapentin relieved hyperalgesia to his feet. Bullae rapidly progressed over his lower extremities, initially concerning for toxin-producing organisms (Figs 3 and 4). Serial debridement demonstrated extensive nonviable tissue; ischemia ultimately necessitated bilateral below-knee amputations.

Trench foot was first described in 1916 during World War I to explain dermatologic patterns observed in soldiers stationed in trenches—this was distinct from frostbite and initially termed “water-bite.”^1^ Modern literature suggests 4 stages: (1) cold exposure, (2) after exposure, (3) hyperemia with hyperalgesia and development of bullae, and (4) after hyperemia.^2^ Diagnosis is clinical. Initial ED treatment includes warmed intravenous fluids, gradual rewarming of limbs, and tetanus booster, with subsequent hospitalization for pain control and wound care and surgical consult if there is evidence of tissue necrosis.^2^

Unhoused individuals suffer exposure injuries with high morbidity, even in temperate climates. Emergency medicine physicians should note that the associated hyperemia and bullae of NFCI may be mistaken for necrotizing soft tissue infection.

## Funding and Support

By *JACEP Open* policy, all authors are required to disclose any and all commercial, financial, and other relationships in any way related to the subject of this article as per ICMJE conflict of interest guidelines (see www.icmje.org). The authors have stated that no such relationships exist.

## Conflict of Interest

All authors have affirmed they have no conflicts of interest to declare.
